# Comparison of Real-time PCR to ELISA for the detection of human cytomegalovirus infection in renal transplant patients in the Sudan

**DOI:** 10.1186/1743-422X-8-222

**Published:** 2011-05-13

**Authors:** Khalid A Enan, Hanna Rennert, Ali M El-Eragi, Abdel Rahim M El Hussein, Isam M Elkhidir

**Affiliations:** 1 Central Laboratory, Ministry of Science and Technology, P.O.Box:7099, Khartoum, Sudan; 2 Department of Pathology and Laboratory Medicine, Weill Cornell Medicine, USA; 3 Central Veterinary Research Laboratories, P.O. Box: 8067, Khartoum, Sudan; 4 Department of Microbiology and Parasitology, Faculty of Medicine, University of Khartoum, P.O. Box: 8067, Khartoum, Sudan

## Abstract

**Background:**

This study was carried out to detect human cytomegalovirus (HCMV) IgG and IgM antibodies using an Enzyme-linked immunosorbent assay (ELISA) in renal transplant patients in Khartoum state, Sudan and to improve the diagnosis of HCMV through the introduction of Real-time Polymerase Chain Reaction (PCR) testing. A total of 98 plasma samples were collected randomly from renal transplant patients at Ibin Sina Hospital and Salma Centre for Transplantation and Haemodialysis during the period from August to September 2006.

**Results:**

Among the 98 renal transplant patients, 65 were males and 33 females. The results revealed that HCMV IgG was present in all patients' plasma 98/98 (100%), while only 6/98 (6.1%) had IgM antibodies in their plasma. HCMV DNA viral loads were detected in 32 patients 32/98 (32.7%) using Real-time PCR.

**Conclusions:**

The HCMV IgG results indicate a high prevalence of past HCMV infection in all tested groups, while the finding of IgM may reflect a recent infection or reactivation. HCMV detection by real-time PCR in the present study indicated a high prevalence among renal transplant patients in Khartoum. In conclusion, the prevalence of HCMV in Khartoum State was documented through detection of HCMV-specific antibodies. Further study using various diagnostic methods should be considered to determine the prevalence of HCMV disease at the national level.

## Background

Human cytomegalovirus (HCMV) is a member of the *Betaherpesvirinae *subfamily which belongs to the family *Herpesviridae *[1]. *Betaherpesviruses*, also includes human herpesvirus 6 (HHV-6) and human herpesvirus 7 (HHV-7), viruses that are more closely related to each other than to other herpesviruses. HCMV is strictly species specific, as are the other cytomegaloviruses. As with all members of the family, the virus can persist in the host in a latent state after primary infection [2]. It is a ubiquitous virus, the seroprevalence of which reportedly varies between 30 and 100% in different countries. Transmission of HCMV can occur vertically or horizontally via direct contact with infectious body fluids or blood. The virus can also be transmitted by blood products or transplanted organs [2]. Although rarely pathogenic in immunocompetent individuals, the virus poses a significant health threat to immunocompromised individuals, and is an important cause of morbidity and mortality, especially in organ allograft and bone marrow transplant patients [3]. When the host immune system is compromised, either through infection- with human immunodeficiency virus (HIV), immunity (neonate), or through iatrogenic means following organ transplantation, the virus is able to exert its full pathogenic potential [2]. Many risk factors for the development of HCMV symptomatic disease have been suggested, and viral load is known to be a major indicator for development of HCMV disease [2].

Diagnosis of HCMV disease is based on clinical symptoms, but the symptoms of HCMV can be confused with those due to Epstein-Barr virus (EBV), and this may lead to difficulties in diagnosis. Laboratory confirmation can be achieved using serological and molecular techniques.

No information is documented about HCMV or related disease prevalence in the Sudan. Hence, the aim of this research work was to generate preliminary information about HCMV infection in renal transplant patients and to establish and improve laboratory diagnosis of HCMV in Sudanese renal transplant patients.

## Results

### Detection of HCMV antibodies

HCMV IgG antibodies were present in the plasma samples of all patients tested, 65 (66.3%) males and 33 (33.7%) females. (Table 1). However, HCMV IgM antibodies were demonstrable in plasma samples of only six of the 98 patients (6.1%) tested. Of these positive patients, 5 (5.1%) were males while only one (1.02%) was a female (Table 1).

**Table 1:** Comparison between ELISA IgG and IgM for the diagnosis of HCMV in plasma samples collected from kidney transplant patients in Khartoum State, Sudan, during the period from August to September, 2006

	Gender	Positive	Negative	Total tested
IgG	Male	**65**	0	**65**
	Female	**33**	0	**33**
IgM	Male	**5**	60	65
	Female	1	32	33

### CMV Real-Time PCR

HCMV DNA was detected in 32/98 (32.7%) of the samples tested, with viral loads ranging from <200 to 42932 copies/mL. In terms of gender; 20 (20.4%) were males while 12 (12.2%) were females.

### Comparison of IgG and IgM ELISA with Real-Time PCR results

The cross-tabulation between real-time PCR and ELISA (IgM and IgG) is shown in (Table 2). A total of 32 patient samples exhibited both IgG by ELISA and DNA by Real-time PCR. Likewise, IgM was detectable by ELISA in plasma samples from two patients with DNA concomitantly demonstrable by Real-time PCR. By comparison, IgG was detected in samples from 66 patients, with no DNA detectable by Real-time PCR. Similarly, IgM was present in four serum samples tested by ELISA, but no DNA was detected by Real-time PCR.

**Table 2:** Cross-tabulation between Real-time PCR and ELISA (IgM and IgG) results for the diagnosis of HCMV in plasma samples collected from kidney transplant patients in Khartoum State, Sudan, during the period from August to September, 2006

			Real-time PCR		
				
			Positive	Negative	Total
ELISA	IgG	Positive	32	66	98
		Negative	0	0	0
		Total	32	66	98
	IgM	Positive	2	4	6
		Negative	30	62	92
		Total	32	66	98

## Discussion

HCMV continue to be a significant cause of morbidity and mortality in immunocompromised patients such as organ or renal transplant recipients [4]. Effective pre-emptive antiviral therapy like gancyclovir and foscarnet depends on detecting HCMV disease at an early stage of infection [5]. Rapid and accurate diagnosis of HCMV infection is an essential part of such case management. Moreover, the quantification of the systemic HCMV load may provide a highly sensitive and specific method to predict which patients will develop HCMV disease.

The present study has focused on the serologic and molecular diagnosis of HCMV in renal transplant patients in Khartoum State in the Sudan. The 100% prevalence of HCMV IgG antibodies found in this study indicates a high rate of past infection, and demonstrates that HCMV infection is common in Sudan. This high prevalence of HCMV may be attributed to the poor socioeconomic status, poor living conditions, and hygienic practices [6]. In contrast to IgG results, IgM antibodies were detected in only 6.1% of the investigated patients. This finding may reflect an active infection [7]. These findings might be considered alarming indicators of disease in such patients.

The prevalence of HCMV infection in the general population of African countries as well as in transplant patients is reportedly high. The seroprevalence has been reported to be 90% in Eritrea [8], 77.6% in Ghana [9], and 36.2% in Egypt [10]. Our results parallel those findings.

Serologic tests results in renal transplant patients can be confused by blood transfusions and/or antibody-based therapies. Furthermore, increases or decreases in antibody levels, in general, do not necessarily support an actual diagnosis of HCMV infection in the immunosuppressed patient populations, due to frequent reactivation of the virus. Therefore, serology has limited diagnostic value in the transplant patient group [11]. A Real-Time PCR method was used in the present study as a highly sensitive and specific method to predict which patients will develop HCMV disease. HCMV by real-time PCR was detected in 32/98 (32.7%) patients, a rate which was clearly higher than the IgM result (6.1%). This might be attributed to the fact that quantification of HCMV DNA is considered both more sensitive and more specific [12].

There was considerable variation in the viral loads in the current study, a function of the sensitivity and specificity of real-time PCR for detection of HCMV DNA as compared to seroloic tests. However, the discrepancy between the obtained negative results using IgM ELISA with the corresponding positive results by real-time PCR may be partially attributable to the time lag between primary infection and IgM antibody production since IgM antibodies may remain undetectable because of delayed seroconversion due to patient treatment with immunosuppressive agents [11]. HCMV DNA was not detected in four patients who were IgM positive. This might be due to the persistence of IgM antibodies for an extended period of time after primary infection, as has been noted in some healthy individuals [11].

In the present investigation, six patients displayed a high viral load (mean value 17532.83 copies/ml) which closely correlated with their clinical status suggestive of active HCMV infection. Two of these patients died within one year. Our results are in agreement with that of Vincent *et. al *[13] who reported that HCMV load in the initial phase of active infection as well as the rate of increase in viral load both correlate with HCMV disease in transplant recipients. The balance of the patients (26) who had lower viral loads (mean value 23.076 copies/ml) were asymptomatic.

## Conclusions

This study was geared to serve as a baseline data analysis for future studies, with a long term goal of introducing preemptive (anti-viral drugs) therapy against HCMV in the Sudan. However, further research work should be carried out to study the epidemiology and to characterize HCMV at the molecular level.

## Methods

### Study population, data and biosample collection

Renal transplant recipients, who were not treated with antiviral therapy, were recruited into this study. These participants were recruited through the Ibin Sina Hospital and Dr. Salma Center for Transplantation and Haemodialysis, Khartoum State, between August and September of 2006. All patients in this study presented to the clinics suffering from a variable spectrum of complaints; i.e., fever, diarrhea, hepatitis, neutropenia and/or thrombocytopenia. Most of the patients presented 2-3 months after kidney transplantation. Case status and clinical information was abstracted by reviewing medical records. The collected data included age, gender, date of transplantation, date of sample collection, and patient location during sample collection. IRB approval for this study was obtained by the Ibin Sina Hospital, Khartoum, Sudan.

A total of 98 blood samples were collected from the patients. Plasma was separated by centrifugation and stored frozen at -20°C until further analyses. Plasma samples were labeled with a unique non-identifying code and express-shipped to New York for CMV DNA analyses.

#### Serology and CMV Real-Time PCR analyses

Commercial ELISA kits (Biokit, Barcelona, Spain) were used to detect HCMV IgG and IgM antibodies according to the procedure described by the manufacturer.

For CMV testing, DNA was extracted from 200 μL of plasma using the QIAamp DNA Extraction kit (QIAGEN, Germantown, MD, USA). For each plasma sample, 5.5 μL (1 mg/mL) Carrier RNA and 10 μL CMV internal control were added to 200 μL of AL lysis buffer following the manufacturer's recommendations. The sample was eluted in 50 μL and 20 μL were used for the assay. CMV viral load testing was carried out using the *artus *CMV PCR assay according to the manufacturer's instructions (QIAGEN). The *artus *CMV™ Master Mix contains reagents and enzymes for the specific amplification of a 105-bp region of the major immediate early antigen. A standard curve was obtained from the quantitation standard (QS) CMV DNA positive controls (CMV TM QS 1-4) provided by the manufacturer. For the PCR amplification, 20 μL of DNA sample eluate was added to 30 μL of the working master mix. The amplicons were then detected by measuring fluorescence, using the ABI PRISM^® ^7900HT Sequence Detection System (Applied Biosystems, Carlsbad CA, USA) with the following amplification conditions: 95°C for 10 min, followed by 45 cycles of 95°C for 15 sec and 55°C for 1 min. At the end of the run, the data were analyzed using the SDS detection software v2.2.2 (ABI). The linear range of the CMV DNA test was from 2.3 to 5.7 log10 copies/mL. Quantitative results were reported if >2.3 log10 copies/mL (200 copies/mL); positive results below 2.3 log10 copies/mL were reported as <200 copies/mL, CMV DNA detected.

## Competing interests

The authors declare that they have no competing interests.

## Authors' contributions

KAE carried out the sample collection, immunoassays, viral load assays, and drafted the manuscript. HR Contributed to the viral load assays and revised the manuscript. AME contributed to the conception and design of the study and acquisition of funding. AE revised the manuscript. IME conceived of the study, and participated in its design and coordination. All authors read and approved the final manuscript.
